# Readiness and Knowledge of Medical Students and Staff in the UAE Toward the Integration of Artificial Intelligence in Medicine

**DOI:** 10.7759/cureus.110639

**Published:** 2026-06-11

**Authors:** Dima Al-Qaimari, Aya Ewida, Omar Abu Eid, Hisham Sinan, Ola Yasir Abdelaziz Osman, Reem Ibrahim Ali Mohamad, Emad A Nosair, Amal Hussein

**Affiliations:** 1 Clinical Sciences, College of Medicine, University of Sharjah, Sharjah, ARE; 2 Basic Medical Sciences, College of Medicine, University of Sharjah, Sharjah, ARE; 3 Family and Community Medicine and Behavioral Sciences, University of Sharjah, Sharjah, ARE

**Keywords:** artificial intelligence, health informatics, medical education, medical faculty, medical students

## Abstract

Background and aim

Artificial intelligence (AI) has become an indispensable component of medical practice. Studies have shown that medical students worldwide are often ill-prepared to take full advantage of these technologies in their future practice due to inadequate training and a shortage of qualified instructors. These factors hinder the adoption of AI-focused curricula; therefore, this study aims to explore medical students’ and faculty’s knowledge and attitudes toward AI applications in the medical field, as well as their readiness for its integration into the medical curriculum, highlighting both the benefits and barriers.

Methods

A cross-sectional study was conducted on medical students (years 1-5) and faculty members from the University of Sharjah College of Medicine (UOSCOM), Sharjah, UAE. They were recruited via convenience sampling between February and March 2023. Students and faculty completed self-administered questionnaires, both online and in person. Analysis was performed using IBM SPSS Statistics for Windows, Version 26 (Released 2018; IBM Corp., Armonk, NY, USA).

Results

Of a total of 413 participants, 74.3% (faculty and students) agreed that the medical field has benefited from AI. Interestingly, 54.3% of students agreed on implementing AI in the curriculum, and 79.3% believe it is necessary, as they see it as a way to empower their careers in the future. However, 51.9% expressed concerns regarding the increase in workload. Of the 45 faculty participants, 66.7% believe that teaching AI would be an addition to their CVs, while 40% disagree that adding the course will increase their workload. Moreover, 77.8% are willing to participate in the course.

Conclusions and recommendations

The findings revealed a general positive perception, with both groups acknowledging the benefits of AI in medicine. Students show awareness of AI applications in education and healthcare, while faculty express willingness to teach AI courses for professional development. However, we recommend that future studies assess students’ baseline computer knowledge and correlate faculty members’ specialty fields with their willingness to teach.

## Introduction

As of 2022, an estimated 1.36 million medical students are enrolled in medical schools worldwide [1], aiming to advance the field of medicine and contribute to innovation within the profession. Artificial intelligence (AI) serves as a crucial link to this future. It is an increasingly recognized element of medicine and is expected to be integrated into routine practice. The potential of AI to revolutionize medical practice is immense, and numerous studies support its integration into medical education. It was shown that AI provided intraoperative assistance via video images and communication technologies, particularly in settings where access to clinics is limited, travel is restricted, or during public health emergencies. Additionally, AI-supported minimally invasive techniques significantly reduced hospital length of stay [2,3]. In today's digitized healthcare environment, the quality of clinical decision-making strongly depends on the accessibility and reliability of the underlying data [4]. As these technologies become increasingly integral to medical practice, evaluating how well medical professionals and undergraduate students are prepared to integrate AI into their practice becomes crucial.

Current and upcoming medical students have had tremendous exposure to digital technologies. This generation is referred to as "digital natives," in contrast to earlier generations, referred to as "digital immigrants" [5]. However, even with this technological familiarity, new generations still require structured guidance to apply these tools safely, ethically, and effectively within clinical contexts.

Despite AI's growing significance in enhancing diagnostic accuracy, enabling personalized treatments, and optimizing healthcare workflows, its integration into medical curricula remains a notable gap. Several barriers have been identified, including traditional curricular structures, accreditation concerns, faculty resistance to change, and prioritization of national licensing examination performance [6]. While biomedical sciences and their interconnectedness with clinical expertise must remain central to medical education, 21st-century medical curricula should also include components that strengthen physicians’ ability to practice effectively in data-rich environments supported by AI technologies [7]. Moreover, a course loses much of its value without competent teachers to guide students through the material. There is a pressing need for capacity-building efforts to expand the number of qualified instructors, as current educators are often overwhelmed with workload demands [8]. Consequently, a dual gap persists in both learner preparedness and educator capacity that we need to address.

Therefore, this study aims to investigate: (1) the sources of information about AI among medical students and faculty, (2) the level of knowledge and attitudes toward AI applications in the medical field, and (3) their readiness for AI implementation in the medical curriculum.

## Materials and methods

Study design

This study employed a quantitative, cross-sectional design to assess the awareness, knowledge, and attitudes of medical students and faculty at the University of Sharjah College of Medicine (UOSCOM), Sharjah, UAE, regarding the integration of AI into the medical curriculum. A cross-sectional design was selected because it allows estimation of prevalence and the exploration of associations at a single point in time.

Study population and sampling

The target population consisted of medical students and faculty members in the UAE, with the accessible population limited to those affiliated with UOSCOM. Inclusion criteria were undergraduate medical students enrolled in years 1-5 and full-time teaching faculty. Exclusion criteria included foundation-year and postgraduate students, as well as adjunct or part-time faculty.

Participants were recruited using convenience sampling. Students were invited to participate via a QR code or a direct survey link, while faculty members were approached in their offices and provided printed copies of the questionnaire. Participation was voluntary for both groups.

Sample size

The required sample size was calculated using a standard single-proportion formula, assuming a 95% confidence interval and a 5% margin of error. Based on a previously reported prevalence of 63.1% awareness [9], the minimum required sample size was estimated at 358 participants. To account for incomplete or missing responses, an additional 10% was added, resulting in a final target sample size of 393 participants.

Research instrument

Two versions of a structured, self-administered questionnaire were developed (Appendix 1): one for students (21 items) and one for faculty (25 items), including demographic information. Each questionnaire was divided into three domains: awareness, attitudes, and perceptions regarding AI integration. Items were primarily close-ended, using five-point Likert scales (1 = strongly disagree, 5 = strongly agree), and multiple-choice questions. Several items were also presented in tabular format, requiring structured responses.

The questionnaire included conditional branching logic: students who answered "yes" to awareness-related questions were directed to follow-up items, while those who answered "no" received an alternative set of questions. A similar structure was used for faculty respondents. The estimated completion time was five to seven minutes for students and six to eight minutes for faculty.

To ensure clarity and face validity, the instrument was piloted among approximately 12 individuals from the College of Medicine prior to data collection. 

Data collection

Data were collected between February and March 2023. Students completed the questionnaire online via Google Forms (Google, Inc., Mountain View, CA, USA), accessed through a QR code or direct link, while faculty members completed paper-based questionnaires distributed by the research team. Informed consent was obtained electronically or in written form prior to participation.

Ethical considerations

Confidentiality and anonymity were maintained by requesting only the last three digits of participants’ phone numbers and the last three letters of their surnames, which were used solely for duplicate identification. All responses were anonymized, and only the research team and supervisor had access to the dataset. Ethical approval was obtained from the Research Ethics Committee at the University of Sharjah. Participants were informed that they could withdraw at any time prior to submission; however, due to anonymization, responses could not be withdrawn after submission.

Data analysis

A total of 368 student responses and 45 faculty responses were included in the final analysis. Data were exported into Microsoft Excel (Microsoft® Corp., Redmond, WA, USA), coded, and analyzed using IBM SPSS Statistics for Windows, Version 26 (Released 2018; IBM Corp., Armonk, NY, USA).

Descriptive statistics were reported as frequencies and percentages. For Likert-scale items, "agree" and "strongly agree" were combined into an "agreement" category, while "disagree" and "strongly disagree" were combined into a "disagreement" category; neutral responses were retained separately. Graphical representations, including bar charts, were generated using Excel.

Bivariate analyses were conducted using the Chi-square test of independence to assess associations between categorical variables. A p-value ≤ 0.05 was considered statistically significant.

## Results

Over six weeks, 377 undergraduate medical students and 45 medical faculty members at UOSCOM responded to the questionnaire. Due to missing data, 368 student responses were valid for statistical analysis, while all 45 faculty responses were included. Of the combined responses, 250 were female (60.5%), and 163 were male (39.5%). The response distribution among medical students was as follows: 28% first-year, 28.5% second-year, 25% third-year, 11.4% fourth-year, and 7.1% fifth-year. Regarding the faculty, the distribution was as follows: 26.7% clinical tutors, 11.1% lecturers, 26.7% assistant professors, 17.8% associate professors, and 17.8% professors.

Table 1 presents descriptive statistics for the questionnaire items regarding the sources of information about AI in medical practice. Most medical students (n = 245, 66.6%) reported learning about AI in medicine through social media, while most faculty members (n = 27, 60%) reported attending conferences as their main source of information. An interesting finding is that a substantial proportion of students (n = 114, 30.9%) learned about AI in high school and in medical curricula (n = 112, 30.4%). In contrast, only (n = 6, 13.3%) faculty members reported acquiring their AI knowledge during their studies. Only a minority of students (n = 31, 8.4%) and faculty (n = 1, 2.2%) reported having no source of information about AI.

**Table 1:** Participants' (n = 413) sources of information about the application of AI in medical practice. The data has been represented as N (%).

	Source of Information About AI	Yes	No
Students (n = 368)	Highschool curriculum	114 (30.9%)	254 (69.0%)
	Medical courses curriculum	112 (30.4%)	256 (69.6%)
	Non-medical courses	74 (20.1%)	294 (79.9%)
	Conferences	37 (10.1%)	331 (89.9%)
	Social media	245 (66.6%)	123 (33.4%)
	Friends/family	173 (47.0%)	195 (53.0%)
	No source of information	31 (8.4%)	337 (91.6%)
Faculty (n = 45)	Study years	6 (13.3%)	39 (86.7%)
	Research	22 (48.9%)	23 (51.1%)
	Conferences	27 (60.0%)	18 (40.0%)
	Social media	24 (53.3%)	21 (46.7%)
	Friends/family	10 (22.2%)	35 (77.8%)
	No source of information	1 (2.2%)	44 (97.8%)

In the second part of the survey, we asked about students’ knowledge of AI use in the medical field (Table 2). Most of our subjects were familiar with AI use in various fields, e.g., medical education (n = 296, 80.4%), medical imaging (n = 286, 77.7%), and surgery (n = 257, 69.8%). On the other hand, only 136 participants (37.0%) were aware of its use in the advising system.

**Table 2:** Students' (n = 368) knowledge of AI use in the medical practice. The data has been represented as N (%).

Field of Medicine	Yes	No
Surgery	257 (69.8%)	111 (30.2%)
Medical imaging	286 (77.7%)	82 (22.3%)
Advising system	136 (37.0%)	232 (63.0%)
Medical education	296 (80.4%)	72 (19.6%)

**Figure 1:**
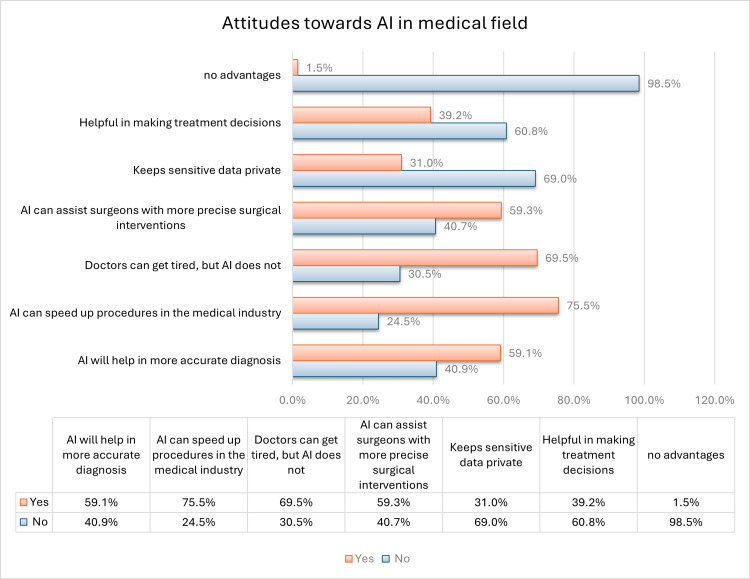
Participants' (n = 413) attitude about the use of AI in medical practice. The data has been represented as %.

Regarding attitudes toward the usability of AI in the medical field, nearly all participants (n = 407, 98.5%) agreed that AI has been helpful in some way. Reported advantages included speeding up medical procedures (n = 312, 75.5%), not getting tired like doctors (n = 287, 69.5%), and improving diagnostic accuracy (n = 244, 59.1%). However, the majority of participants (n = 285, 60.8%) did not believe that AI would be useful in facilitating treatment decisions. In addition, a majority of participants (n = 323, 69.0%) reported concerns that AI systems may not adequately ensure patient data privacy (Figure 1).

Lastly, we investigated students’ and faculty’s attitudes toward integrating AI into the medical curricula. Nearly half of the students (n = 158, 42.9%) agreed and demonstrated familiarity with AI-related terms, 84 (22.8%) disagreed, and 126 (34.2%) were neutral. On the other hand, faculty demonstrated a higher level of agreement: 26 (57.7%) agreed, 5 (11.1%) disagreed, and 14 (31.1%) remained neutral. An overwhelming majority of both students (n = 200, 54.3%) and teaching staff (n = 30, 66.7%) believe that incorporating AI into the education system will empower their careers and résumés. Likewise, the majority of students (n = 292, 79.3%) and faculty (n = 35, 77.8%) expressed willingness to adopt the course into the curriculum. Although 191 (51.9%) students are concerned that the new AI course would increase their workload, only 12 (26.7%) faculty members shared this concern (Table 3).

**Table 3:** Participants' (n = 413) readiness towards implementation of AI into the medical curriculum. The data has been represented as N (%).

Readiness Towards Curriculum Implementation		Disagree	Neutral	Agree
Familiar with AI terms	Students	84 (22.8%)	126 (34.2%)	158 (42.9%)
	Faculty	5 (11.1%)	14 (31.1%)	26 (57.7%)
The medical field has benefited from AI	Common	30 (7.3%)	76 (18.5%)	307 (74.2%)
Will empower your career/résumés	Students	56 (15.2%)	112 (30.4%)	200 (54.3%)
	Faculty	4 (8.9%)	11 (24.4%)	30 (66.7%)
Will increase your workload	Students	78 (21.2%)	99 (26.9%)	191 (51.9%)
	Faculty	18 (40.0%)	15 (33.3%)	12 (26.7%)
Willing to take/teach a course	Students	76 (20.7%)	-	292 (79.3%)
	Faculty	10 (22.2%)	-	35 (77.8%)

A Chi-square test (p < 0.05) showed a statistically significant relationship between students’ beliefs about the necessity of a course on the applications of AI in the medical curriculum and (1) the importance of AI (χ² = 8.046, p = 0.018) and (2) having reliable knowledge sources (χ² = 6.380, p = 0.012). However, the relationship with the other two determinants, e.g., gender and medical year, showed no significant correlation (Table 4).

**Table 4:** Relationship between the students' gender, year of study, AI importance scale, and reliability of knowledge source, and the belief in the necessity of implementing an AI course into the curriculum. Data are presented as N (%). Chi-square test (χ²) was used. A p-value < 0.05 was considered statistically significant.

Do you believe it is necessary to have a course about applications of Artificial Intelligence in the medical curriculum?							
Variable	Total	Yes		No		χ² value	p-value
		N	%	N	%		
What medical year are you in?							
Year 1	103	79	76.7%	24	23.3%	1.518	0.824
Year 2	105	87	82.9%	18	23.7%		
Year 3	92	73	79.3%	19	20.7%		
Year 4	42	32	76.2%	10	23.8%		
Year 5	26	21	80.8%	5	19.2%		
Gender							
Male	141	115	81.6%	26	18.4%	0.683	0.409
Female	227	177	78.0%	50	22.0%		
Importance of AI Scale							
1 / 2	44	29	65.9%	15	34.1%	8.046	0.018
3	104	79	76.0%	25	24.0%		
4 / 5	220	184	83.6%	36	16.4%		
Reliable Source of Knowledge							
Reliable	239	199	83.3%	40	16.7%	6.380	0.012
Unreliable	129	93	72.1%	36	27.9%		

## Discussion

To our knowledge, this is the first study in the Arab region to investigate the knowledge and attitudes of medical students and teaching faculty toward the integration of AI in medical education. The current study shows that the majority of students and faculty have a high level of knowledge about AI, with 66.6% reporting that they acquired this information from mainstream media rather than university teaching. Pinto Dos Santos et al., however, identified an overall low level of knowledge among medical students regarding AI, despite knowledge acquisition from similar sources. They also highlighted that students who were more knowledgeable about AI were less afraid of working with technology [9]. Since our cohort demonstrates greater knowledge of AI, this suggests a promising, confident future generation of physicians ready to embrace technology in their future practice.

It is now widely accepted that AI will likely have a profound impact on the future practice of radiology and other medical fields. In our cohort, 77.7% of students were aware of the use of AI in radiology. This aligns with findings from Sit et al., in which 98.5% agreed that AI will play an important role in healthcare, and nearly half of the students were less likely to consider a career in radiology because of the perceived success of AI [10]. An adverse impact of AI advancement on radiology recruitment was previously demonstrated in a Canadian cohort (N = 322), in which one-sixth of medical students interested in radiology were discouraged from applying for a residency position. That study found that students were more concerned about the displacement of radiologists than their replacement, potentially reducing workforce demands [11].

Although our cohort believed that AI would help achieve more accurate diagnoses, the majority did not believe it would assist in making accurate treatment decisions and expressed concerns regarding patient data privacy. AI possesses remarkable computational capacity, enabling rapid analysis of large datasets. However, it should be viewed as a supportive tool that enables physicians to provide enhanced, personalized care to their patients, rather than an autonomous decision-maker. Physicians define the operational boundaries within which AI functions. Moreover, medical practice includes much more than scientific reasoning alone. Patient care is dependent on health insurance systems, socio-economic factors, and cultural beliefs - dimensions that are not encoded in AI algorithms [12].

In our study, 42.9% of students and 57.7% of faculty reported familiarity with AI-related terms, similar to findings from Pelita Harapan University, where medical students demonstrated limited knowledge of AI, with only 38.5% of students indicating familiarity with the concept [13]. Our data indicate that faculty members demonstrate greater familiarity with AI than students, possibly reflecting increased professional exposure and engagement with technological developments in medical education. This observation is consistent with previous research showing higher AI awareness among faculty, reinforcing the need for greater structured exposure to AI among students [14].

While 51.9% of students believed that AI integration would increase their workload, only 26.7% of faculty shared this concern. This interesting disparity suggests that students may perceive AI-related content as an added academic burden, whereas faculty may view it as an opportunity to enrich the curriculum. Similar findings were reported by Lugito et al., where students expressed concerns about the additional workload, while faculty largely supported AI integration without anticipating a substantial increase in burden [13]. In contrast, other studies have shown fewer workload concerns among healthcare professionals, including medical students, possibly reflecting variations in institutional frameworks or curriculum design [15]. The few universities that currently offer AI courses utilize diverse teaching strategies, including interactive lectures, small group discussions, e-modules, and elective courses. Curriculum overload can be minimized and the clinical applications of AI can be emphasized by incorporating AI into existing courses [16].

The source of AI knowledge also significantly impacts students’ attitudes toward AI education (p = 0.012). Students who acquired AI knowledge from reliable sources such as academic journals and structured educational programs were more supportive of AI course inclusion (83.3%) compared to those relying on less formal sources (72.1%). This underscores the critical role of providing accurate, accessible, high-quality AI education resources in shaping informed and constructive attitudes.

A significant proportion of both students (54.3%) and faculty (66.7%) believed that AI integration would enhance their careers and professional profiles. Consequently, the majority of respondents (79.3% of students and 77.8% of faculty) expressed a strong willingness to incorporate AI-related courses into medical curricula. Although the gender distribution was not statistically significant (p = 0.409), slightly greater support was observed among male students. Similar trends have been reported across multiple previous studies, where male participants demonstrated greater interest in technology and AI and where both students and faculty acknowledged the career advantages associated with AI competency [13-15].

This consensus emphasizes the growing recognition of AI as a crucial skill for future healthcare professionals in enhancing their clinical and academic competencies. Despite concerns about workload and data privacy, there is strong institutional support for AI integration. This positive outlook presents a valuable opportunity for medical schools to develop structured AI curricula and workshops that promote innovation and continuous professional development. By responding to this momentum, educational institutions can ensure that future medical professionals are well-equipped to integrate AI responsibly and effectively into healthcare delivery.

Limitation

The baseline level of students’ understanding of fundamental computational principles should be systematically assessed before implementing a structured course into the medical curriculum. Without this assessment, curriculum design may not adequately align with the learners’ needs. Additionally, faculty willingness to teach AI-related content should be examined in relation to their specialty, prior exposure to AI, and existing workload, as these factors may partly explain the negative views observed in some responses. Failure to account for these variables may limit the accurate interpretation of faculty attitudes.

This study was conducted at a single institution, which may limit its generalizability to other medical schools or national contexts. Institutional culture, technology, and curriculum structure may differ substantially across settings.

A limitation of this study is the use of mixed data collection methods, as faculty participants were given paper-based questionnaires, while students responded via an online platform. This approach was adopted to improve faculty participation and accommodate varying preferences and accessibility. Although this may have introduced minor mode-related differences in response behavior, it is unlikely to have significantly affected the overall results.

Furthermore, participation in the survey was voluntary, introducing the possibility of self-selection bias. It is possible that students and faculty with pre-existing interest in AI and advanced medical technology were more inclined to participate, potentially leading to an overestimation of positive attitudes toward AI integration. The cross-sectional design also limits the ability to establish causal relationships between knowledge levels, information sources, and attitudes.

Finally, although the questionnaire was piloted to ensure clarity and face validity, formal psychometric validation, including assessment of internal consistency reliability (e.g., Cronbach’s alpha), was not performed. This may affect the strength of measurement validity and should be considered when interpreting the results.

Future directions

In order to improve external validity and facilitate comparisons across various educational systems, we recommend conducting multi-institutional studies and cross-national investigations to further assess readiness and to inform the development of evidence-based recommendations for dedicated task forces aimed at responsibly integrating AI technologies into medical curricula. Longitudinal designs are then needed to evaluate changes in AI knowledge and attitudes following this implementation. A deeper understanding of the perceived obstacles, enablers, and cultural factors influencing the adoption of AI in medical education may also be achieved by incorporating qualitative approaches, such as focus groups or in-depth interviews. Ultimately, the results generated from such studies can guide the creation of competency-based, standardized AI curricula that are both flexible enough to accommodate local institutional contexts and compliant with international medical education frameworks.

## Conclusions

AI has undergone significant evolution to its current state. As technologies continue to advance, medicine stands on the threshold of a new era characterized by the integration of AI into routine academic and clinical practice. This study provides critical insights into current knowledge of AI applications and attitudes toward its integration into the curriculum among medical students and faculty at the University of Sharjah. The finding that a higher percentage of students than faculty rely on less reliable sources of information underscores the need for more structured and credible educational initiatives. Despite limited formal exposure to AI literacy within the curriculum, there is a clear consensus regarding the importance of incorporating AI technologies into medical training. Both students and faculty demonstrate strong interest in AI-related topics and express predominantly positive attitudes toward its role in medicine.

Educators should address students’ concerns regarding increased workload by developing innovative curricular models that integrate AI literacy and practical application into existing learning frameworks rather than adding standalone burdens. Notably, participants demonstrated strong enthusiasm for integrating AI into the curriculum, with both groups expressing willingness either to undertake or deliver the course. Collectively, these findings highlight the urgency of developing a well-structured curriculum that addresses the technical, practical, and ethical dimensions of AI in order to equip the next generation of physicians with the competencies required to deliver the best patient care in an increasingly AI-driven healthcare future.

## Appendices

Appendix 1

**Table 5:** Questionnaire used in the study. This questionnaire was developed by the authors of this study after reviewing the literature.

Section Name	Item Number & Question Type	Questions/Instructions	Choices
Demographics (Students)	1. Single choice	Are you a medical student or a medical faculty member?	Medical student / Medical faculty member
	2. Single choice	Gender	Male / Female
	3. Single choice	Age group	≤20 years / 21-25 years / ≥26 years
	4. Single choice	Year of study	Year 1 / Year 2 / Year 3 / Year 4 / Year 5
Knowledge & Awareness (Students)	5. Likert scale	Familiarity with AI-related terminology	Strongly agree / Agree / Neutral / Disagree / Strongly disagree
	6. Linear scale (1-5)	Importance of AI in the medical field	1 (Not important) - 5 (Extremely important)
	7. Multiple choice	Where is AI being used?	Surgeries / Radiology (CT, MRI) / Educational software / Student advising systems
	8. Multiple choice	Source of AI knowledge	High school curriculum / Medical curriculum / Non-medical courses / University workshops / Social media / Friends & family / Other / No information
Attitudes (Students)	9. Likert scale	AI has benefited the medical field	Strongly agree / Agree / Neutral / Disagree / Strongly disagree
	10. Likert scale	AI has better diagnostic capability than doctors	Strongly agree / Agree / Neutral / Disagree / Strongly disagree
	11. Likert scale	AI could replace your future job	Strongly agree / Agree / Neutral / Disagree / Strongly disagree
	12. Likert scale	You will use AI in medical decision-making	Strongly agree / Agree / Neutral / Disagree / Strongly disagree
	13. Multiple choice	Advantages of AI	Accurate diagnosis / Faster procedures / No fatigue / Surgical precision / Data privacy / Treatment decisions / Reduced errors / Other / No advantages
	14. Likert scale	Lack of emotional intelligence is concerning	Strongly agree / Agree / Neutral / Disagree / Strongly disagree
	15. Likert scale	AI is unreliable in unpredictable situations	Strongly agree / Agree / Neutral / Disagree / Strongly disagree
Curriculum (Students)	16. Single choice	Need for AI course	Yes / No
	17. Single choice	Preferred course type	Elective / Mandatory
	18. Open-ended	If no, specify why	Free text
	19. Likert scale	AI course increases workload	Strongly agree / Agree / Neutral / Disagree / Strongly disagree
	20. Likert scale	AI improves future career	Strongly agree / Agree / Neutral / Disagree / Strongly disagree
Knowledge & Awareness (Faculty)	21. Likert scale	Familiarity with AI in medicine is good	Strongly agree / Agree / Neutral / Disagree / Strongly disagree
	22. Likert scale	Comfort with AI terminology	Strongly agree / Agree / Neutral / Disagree / Strongly disagree
	23. Multiple choice	Source of AI knowledge	Education / Conferences / Social media / Research / Friends & family / Other / No information
	24. Single choice	Experience teaching AI	Yes / No
Attitudes (Faculty)	25. Likert scale	AI has benefited medicine	Strongly agree / Agree / Neutral / Disagree / Strongly disagree
	26. Likert scale	AI has better diagnostic ability than doctors	Strongly agree / Agree / Neutral / Disagree / Strongly disagree
	27. Likert scale	AI could replace doctors	Strongly agree / Agree / Neutral / Disagree / Strongly disagree
	28. Multiple choice	Advantages of AI	Accurate diagnosis / Faster procedures / No fatigue / Surgical precision / Data privacy / Treatment decisions / Other / No advantages
	29. Likert scale	Lack of emotional intelligence is concerning	Strongly agree / Agree / Neutral / Disagree / Strongly disagree
	30. Likert scale	AI unreliable in unpredictable situations	Strongly agree / Agree / Neutral / Disagree / Strongly disagree
Curriculum (Faculty)	31. Single choice	Recommend AI course	Yes / No
	32. Single choice	Willing to teach AI	Yes / No
	33. Likert scale	AI increases workload	Strongly agree / Agree / Neutral / Disagree / Strongly disagree
	34. Likert scale	AI improves CV	Strongly agree / Agree / Neutral / Disagree / Strongly disagree
	35. Single choice	Best year for the AI course	Year 1 / Year 2 / Year 3 / Year 4 / Year 5
	36. Single choice	AI course burdens students	Yes / No
	37. Linear scale (1-5)	AI prepares students for a career	1-5 scale
	38. Single choice	AI affects employability	Yes / No
